# Preparation of Sludge-Derived Activated Carbon by Fenton Activation and the Adsorption of Eriochrome Black T

**DOI:** 10.3390/ma12060882

**Published:** 2019-03-16

**Authors:** Haifeng Wen, Daofang Zhang, Lin Gu, Haixiang Yu, Minmin Pan, Yuanxing Huang

**Affiliations:** School of Environment and Architecture, University of Shanghai for Science and Technology, Shanghai 200093, China; hai23@126.com (H.W.); lin_gu@hotmail.com (L.G.); xray1201@163.com (H.Y.); claire_panmm@163.com (M.P.); huangyuanxing@usst.edu.cn (Y.H.)

**Keywords:** Fenton, sludge-derived activated carbon, adsorption, Eriochrome Black T

## Abstract

Sludge-derived activated carbon (SAC) was prepared by Fenton activation and calcination, and used as adsorbent to eliminate Eriochrome Black T (EBT) dye from aqueous media. The characterization results indicated that the produced SAC had a porous structure, high specific surface area, and abundant functional groups on its surface. The adsorption process was affected by pH, adsorbent dosage, time, and temperature. The adsorption capacity increased with temperature, and the highest adsorption capacity reached 178.2 mg·g^−1^ in 48 h at 318 K and pH 6. The results of the adsorption isotherm, kinetic, and thermodynamic analyses revealed that the adsorption of EBT onto SAC was naturally endothermic and spontaneous, involved both physical and chemical processes, and belonged mostly to the multilayer type of adsorption.

## 1. Introduction

**Figure 1:**
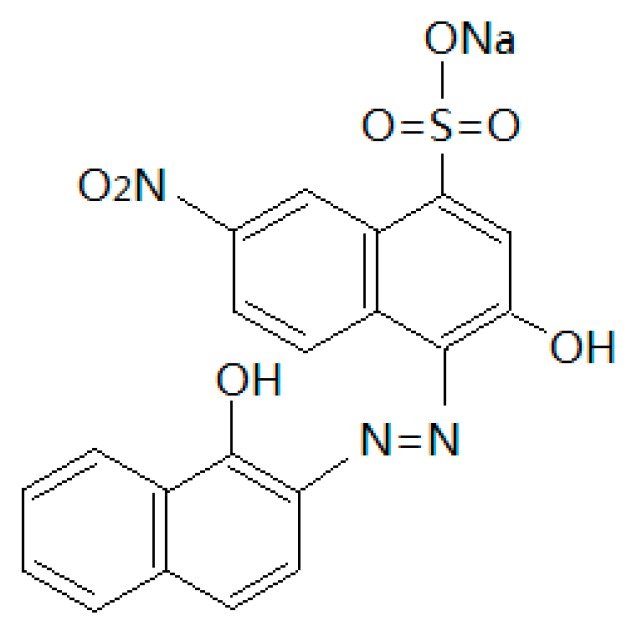
The chemical structure of Eriochrome Black T (EBT).

Dyeing wastewater is characterized as having complex components and high chrominance. It contains various toxic and highly non-degradable organic compounds and has very high chemical oxygen demand (COD), biological oxygen demand (BOD), and high acidity or alkalinity [1,2]. Dyeing wastewater is regarded as one of the hardest-to-treat forms of industrial wastewater [3]. About 70% of the dyeing wastewater utilized worldwide by dyeing industries is composed of azo dyes that have one or more azo bonds, which are toxic to many organisms and might cause the direct destruction of creatures in water [2,4]. Eriochrome Black T (EBT) dye belongs to the azo dyes, which have potential environmental hazards and constitute a direct threat to human health [5]. EBT is often used as an indicator in complexometric titrations; the application of EBT in industrial processes produces large amounts of waste effluent. Figure 1 shows the chemical structure of EBT.

For resistant dyeing wastewater, the commonly employed treatment methods include electrochemical treatment and advanced oxidation processes (AOPs), such as ozonation and photocatalysis [6,7,8]. However, these technologies are generally cost-inefficient and complicated to execute. Among many methods, adsorption is one of the most simple, efficient and comparable low-cost processes applied in removing the color, heavy metals, and other inorganic and organic contaminants from wastewater [9,10].

On the other hand, the disposal of huge amounts of sewage sludge from wastewater treatment plants is also becoming a growing problem [11]. The reduction, stabilization, innocuity treatment and resource utilization of sewage sludge is now an urgent issue. Sewage sludge is usually rich in carbonaceous content that makes it possible for them to be converted into activated carbon (AC) and applied as an adsorbent in wastewater purification [12]. It is difficult to dewater sewage sludge because of its high organic content, thus bringing problems for its transportation, storage, and disposal, and causing its management to be expensive [13]. To enhance the performance of conditioning and dewatering, many approaches have been tried. AOPs, especially the Fenton reaction, have been widely investigated in this regard [14]. The Fenton reaction is a catalytic process based on the simultaneous use of iron salts and hydrogen peroxide under acidic conditions. It involves the formation of large quantities of hydroxyl radicals that can destroy organic matter in a sewage sludge, thus improving the dewaterability of the sewage sludge [15].

Currently, the studies on the activation of SAC using the Fenton process, as well as the use of Fenton-activated SAC for EBT adsorption are limited, and this topic still deserves deeper research. Thus, in this paper, the Fenton reaction was employed to activate the sewage sludge, and the activated sewage sludge was then subjected to dewatering, drying, and high-temperature carbonization to produce sludge-derived activated carbon (SAC). The produced SAC was characterized. The removal of EBT dye from aqueous solution by the produced SAC as adsorbent was investigated. Experimental data were applied to the Langmuir and Freundlich models. Kinetic studies were carried out using pseudo-first- and pseudo-second-order equations, and the adsorption mechanism was also analyzed.

## 2. Materials and Methods

### 2.1. Materials and Reagents

The FeSO_4_·7H_2_O, H_2_O_2_ solution, NaOH, H_2_SO_4_, HCl and EBT were of analytical grade and purchased from Sinopharm Chemical Reagent Co., Ltd. (Shanghai, China). Deionized water was used to prepare all solutions.

The sewage sludge was collected from a secondary clarifier from Shanghai Songshen Water Environment Purification Co., Ltd. (Shanghai, China). The properties of the sewage sludge are shown in Table 1. The element composition was analyzed by an elemental analyzer (MACRO, Elementra, Germany).

### 2.2. Preparation and Characterization of the SAC

The preparation of the SAC was based on the method introduced by [16]. A total of 30% (v/v) H_2_SO_4_ solution was used to adjust the pH of the sewage sludge (2 L) to 3.0, then Fenton’s reagent was added, where the H_2_O_2_ concentration was 3.75% (v/v), and H_2_O_2_/Fe^2+^ mass ratio was 7.5:1. After reacting for 2.0 h at room temperature, 5 mol·L^−1^ NaOH was added to adjust the pH of the mixture to 10.0–11.0. The mixture was dewatered by centrifugation, dried at 105 °C for 24 h, grounded and sieved through 100 mesh, then the powder was pyrolyzed at a temperature of 600 °C for 2 h under the protection of N_2_. The pyrolyzed samples were washed in simmering 10% (w/w) HCl for 30 s, then rinsed with deionized water until neutral, and dried at 60 °C to obtain SAC. The obtained SAC was denoted by F-SAC. A parallel SAC sample was produced without the activation of Fenton’s reagent, and denoted by P-SAC.

The SAC was characterized by X-ray diffractometry (XRD) (D8 ADVANCE X, Bruker, Karlsruhe, Germany) with Cu-Kα radiation (40 kV, 40 mA) in the 2θ range of 5–80°, Fourier transform infrared spectroscopy (FT-IR) with a spectrophotometer (PerkinElmer, Waltham, MA, USA), X-ray photoelectron spectroscopy (XPS) (SCALLB 250 XI, Thermo Scientific, Waltham, MA, USA), and scanning electron microscopy (SEM, 230, FEI, Hillsboro, OR, USA). Specific surface areas were measured at a liquid nitrogen temperature of 77 K by a surface area analyzer (Autosorb-iQ, Quantachrome, Boynton Beach, FL, USA) using the Brunauer-Emmett-Teller (BET) method.

### 2.3. Adsorption of EBT by the SAC

The tests of EBT sorption on the SAC were performed under several different conditions. The aqueous EBT concentration was determined by a UV-vis spectrophotometer (UV-2600, Shimadzu, Japan) using a wavelength of 534 nm.

The EBT removal percentage (%) was determined by Equation (1):

(1) $$R=\frac{\left(C_{0}-C_{t}\right)}{C_{0}}\times 100\%$$

where C_0_ is the initial EBT concentration (mg·L^−1^), and C_t_ (mg·L^−1^) is the EBT concentration at time t.

The quantity of adsorbed EBT was determined by Equation (2):

(2) $$q_{t}=\frac{\left(C_{0}-C_{t}\right)V}{m}$$

where q_t_ (mg·g^−1^) is the amount of EBT adsorbed at time t; m (g) is the mass of SAC; and V (L) is the solution volume.

#### 2.3.1. Effect of pH on the Adsorption of EBT by the SAC

The pH-effect experiments were performed with 3.00 g·L^−1^ SAC suspended in 8 beakers of the 50 mL solution containing 500 mg·L^−1^ EBT, with the pH set at 2.0–12.0. The mixtures were equilibrated at a temperature of 298 K on a shaker for 48 h. After adsorption the mixture was filtered by a polyethersulfone membrane with a pore size of 0.45 μm to measure the aqueous EBT concentration. Based on the results we determined the optimal pH.

#### 2.3.2. Effect of the Initial EBT Concentration on the Adsorption of EBT by the SAC

The initial EBT concentration-effect experiments were performed with a series of EBT aqueous solutions (concentration: 100.0–1200.0 mg·L^−1^, volume: 50 mL). The pH of the solutions were adjusted to be optimal, then the solutions were added to 3.00 g·L^−1^ SAC, and the mixtures were equilibrated at a temperature of 298 K on a shaker for 48 h, taking water samples at regular intervals, measuring the concentration of the EBT aqueous solution, and calculating the quantity of adsorbed EBT.

#### 2.3.3. Adsorption Kinetics Studies

The adsorption kinetics studies were performed with a series of EBT aqueous solutions (concentration: 500.0 mg·L^−1^, volume 50 mL) and 3.00 g·L^−1^ SAC under the optimal pH. The mixtures were equilibrated on a shaker for 48 h at temperatures of 288 K, 298 K, 308 K, and 318 K, taking water samples at regular intervals to measure the aqueous EBT concentration.

The EBT adsorption kinetics could be simulated by the Lagrangian first-order model shown in Equation (3) and the pseudo-second-order reaction model shown in Equation (4):

(3) $$\ln\left(q_{e}-q_{t}\right)={\mathrm{lnq}}_{e}-k_{1}$$

(4) $$\frac{t}{q_{t}}=\frac{1}{k_{2}{\left(q_{e}\right)}^{2}}+\frac{t}{q_{e}}$$

where k_1_ (min^−1^) and k_2_ (g·mg^−1^·min^−1^) are the rate constants of adsorption, and q_e_ is the mass of EBT adsorbed per unit mass of SAC at equilibrium (mg·g^−1^).

#### 2.3.4. Adsorption Isotherm Studies

Adsorption isotherms were fitted using the Langmuir Equation (5) and the Freundlich Equation (6) models:

(5) $$\frac{c_{e}}{q_{e}}=\frac{1}{K_{L}q_{m}}+\frac{c_{e}}{q_{m}}$$

(6) $${\mathrm{lnq}}_{e}={\mathrm{lnK}}_{F}+\frac{1}{n}{\mathrm{lnc}}_{e}$$

where c_e_ is the equilibrium concentration of EBT (mg·L^−1^), q_m_ is the maximum adsorption capacity to form a monolayer on the adsorbent surface (mg·g^−1^), K_L_ is the empirically derived Langmuir constant (L·mg^−1^), K_F_ is the Freundlich capacity factor (L^1/n^·mg^(1−1/n)^·g^−1^), and 1/n is Freundlich intensity parameter [17].

## 3. Results and Discussion

### 3.1. Characterization of the SAC

**Figure 2:**
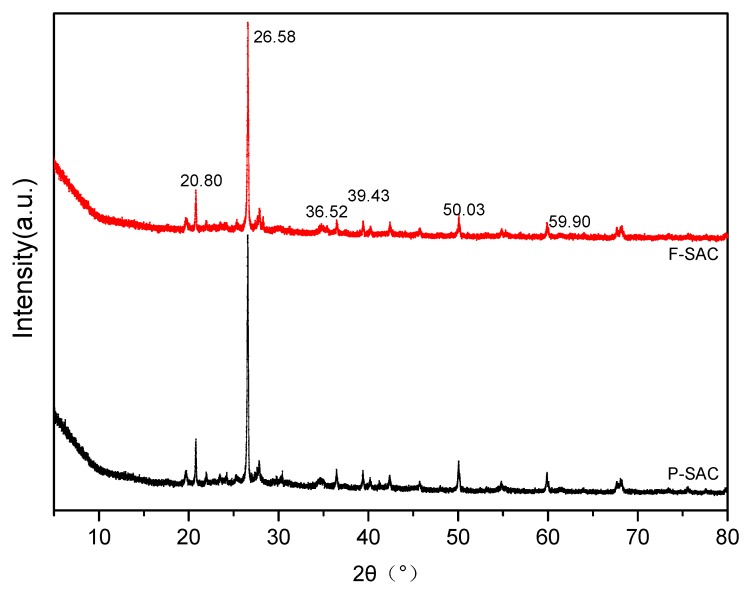
X-ray diffractometry (XRD) of Fenton-activated sludge-derived activated carbon (F-SAC) and untreated SAC (P-SAC).

Figure 2 shows the XRD patterns for the prepared F-SAC and P-SAC. The sharp diffraction peaks at 2θ = 26.58° and 50.03° correspond to the microcrystalline crystal plane (002) and the microcrystalline crystal plane (100), respectively, which indicates a graphite-like structure of the carbon [18]. F-SAC and P-SAC had similar patterns, which indicated their similar structure and composition. With the activation by the Fenton reagent, the peak intensity of F-SAC dropped slightly compared to P-SAC, which might be due to partial destruction of the carbon structure. The small diffraction peaks at 2θ = 20.80°, 36.52°, 39.43°, and 59.90° are the characteristic reflections of SiO_2_.

**Figure 3:**
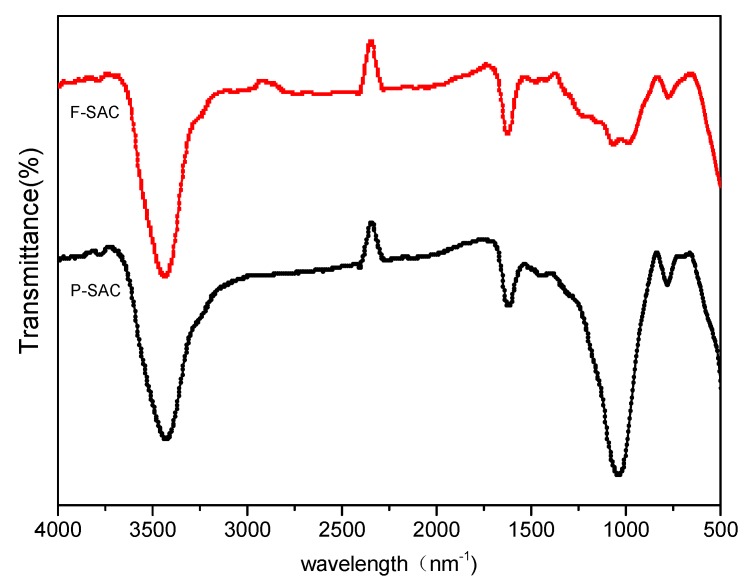
Fourier transform-infrared (FT-IR) spectra of F-SAC and P-SAC.

The FT-IR spectra of F-SAC and P-SAC are shown in Figure 3. The peak near 800 cm^−1^ was from the functional group of aromatic C–H bonds. The band at 1050 cm^−1^ indicated the stretching of Si–O–C and Si–O–Si in the prepared SAC, or the telescopic vibration of aromatic hydrocarbons [18]. The peak transmittance at 1050 cm^−1^ of F-SAC was weaker than that of P-SAC. The peak at 1600 cm^−1^ could be assigned to oxygen-containing functional groups such as C=O, O=C–O, or –COOH. The wide peak at 3300–3500 cm^−1^ indicated the presence of –NH_2_ and –OH [11,19].

**Figure 4:**
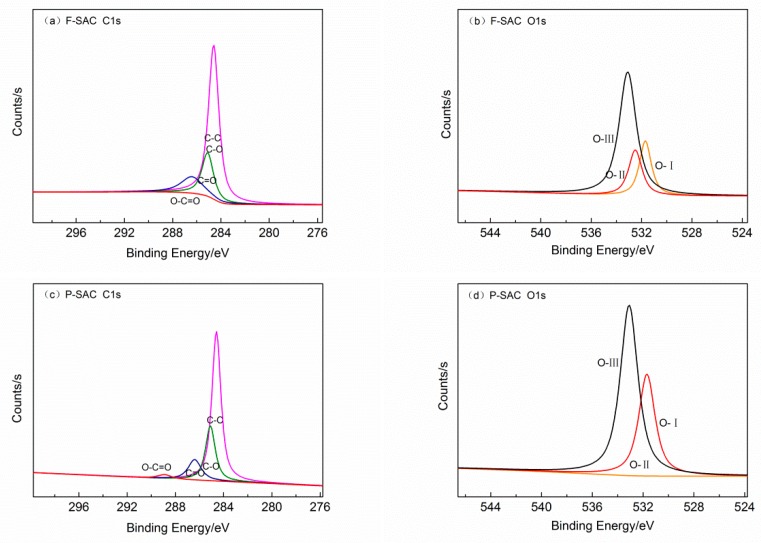
X-ray photoelectron spectroscopy (XPS) spectra of F-SAC and P-SAC. (**a**) F-SAC C1s; (**b**) F-SAC O1s; (**c**) P-SAC C1s; (**d**) P-SAC O1s.

In Figure 4 four peaks can be identified in the C1s region of the SAC samples, 284.6 ± 0.2, 285.1 ± 0.2, 286.4 ± 0.2, and 288.9 ± 0.2 eV, which correspond to C–C, C–O, C=O, and O–C=O, respectively [19]. The proportion of C–C was the highest, with C–O and C=O accounting for a smaller proportion, while that of O–C=O was the smallest. The O1s region of SAC samples could be divided into the following three peaks: 530.3 ± 0.2 (O–H), 531.7 ± 0.2 (–C=O), and 533.1 ± 0.2 eV (C–O–C). It was observed that the O–H content of F-SAC increased, while the C–O–C content decreased.

**Figure 5:**
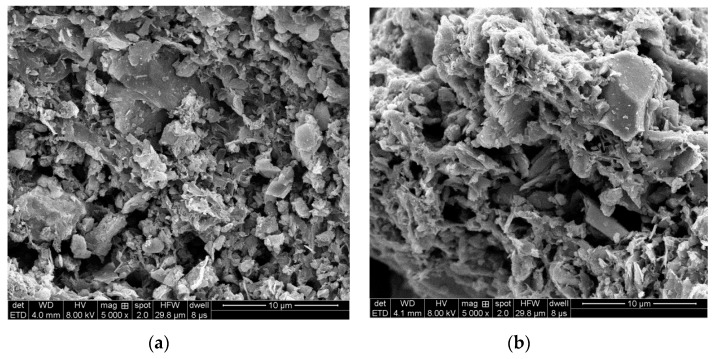
Scanning electron microscope (SEM) images of (**a**) F-SAC and (**b**) P-SAC.

The SEM micrographs shown in Figure 5 display the porous structure of F-SAC and P-SAC. It seems that the tiny pores of F-SAC were more uniform and abundant. According to the test result for nitrogen adsorption, the specific surface area of F-SAC was 172.8 m^2^·g^−1^, which was much higher than that of P-SAC, the specific surface area of which was only 92.6 m^2^·g^−1^, as indicated in Table 2. This result was consistent with that of the SEM, and it implies that the treatment with Fenton’s reagent improved the adsorption function of SAC.

According to the research carried out by Mo et al., the Fenton reaction could collapse the sludge floc and release the bound water rapidly by partially oxidizing and destroying the organic components [20]. The Fe^3+^ generated from Fenton’s reagent acts as a coagulant to agglomerate smaller sludge particles into larger, denser particles with less bound water [21]. During the disintegration and recombination of sludge particles, Fe ions were dispersed and deposited into the inner layer and embedded into the carbon matrix. In the subsequent thermal pyrolysis, the iron was reduced by carbon, which led to the release of volatile gases and the development of pore structures. The formed pore walls were typically made up of ashes like magnetite, alumina, and silica [22]. Furthermore, the presence of metallic iron was expected to be active in breaking C–C and C–H bonds to cause the biomass gasification and the pore structure formation [23].

### 3.2. Adsorption of EBT by the SAC

#### 3.2.1. Effect of pH on Adsorption

**Figure 6:**
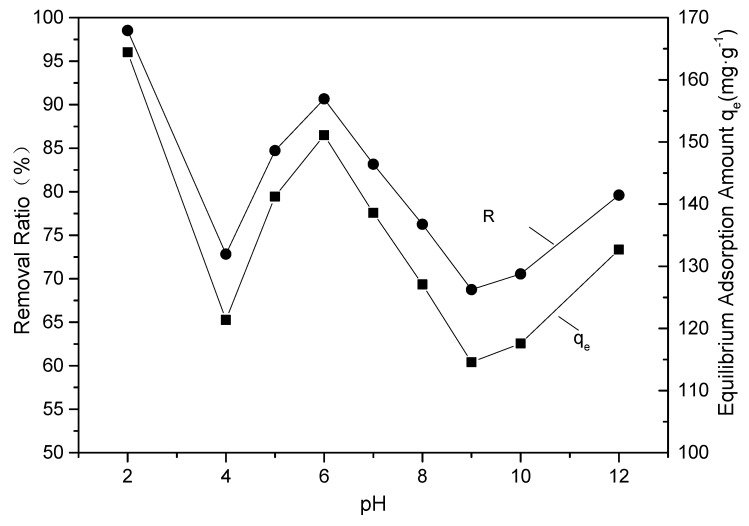
The effect of pH on adsorption of EBT by F-SAC.

The factors that controlled the adsorption process included pH, adsorbate content, the adsorbent properties, and temperature, among others. The results in Figure 6 show that pH played an important role in the adsorption of EBT dye by F-SAC. Given that that solution pH might affect the SAC surface binding sites; surface charge; and chemical state, such as the degree of ionization of EBT, the effect of pH on the adsorption process was complex [24]. The highest EBT removal (98.63%) and the highest adsorption capacity (164.38 mg·g^−1^) were obtained at pH 2. As the pH increased to 4, the EBT removal and adsorption capacity decreased to a minimum, and then increased to 90.66% and 151.10 mg·g^−1^ at pH 6. The lowest EBT removal (68.74%) and adsorption capacity (114.57 mg·g^−1^) occurred at pH 9. For practical operation, strongly acidic conditions and strongly basic conditions were usually inconvenient, thus a moderate pH of 6 would be optimal. These results are consistent with the findings of de Luna, et al., that at acidic pH the functional groups of F-SAC become protonated and enhance the anionic EBT adsorption through electrostatic attraction [2]. The good removal capacity at pH 6 indicated that electrostatic interaction was not the only mechanism for EBT adsorption. SAC might interact with EBT molecules via hydrogen bonding and hydrophobe-hydrophobe mechanisms [25].

#### 3.2.2. Effect of the Initial EBT Concentration on the Adsorption and Adsorption Isotherm

**Figure 7:**
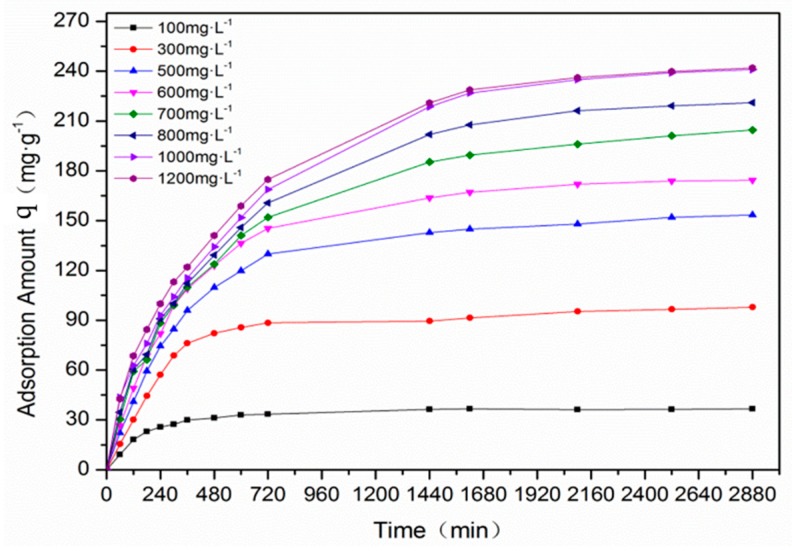
The effect of the initial EBT concentration on the adsorption of EBT by F-SAC.

As reported in some literature, increasing the initial concentration of adsorbate could increase its removal efficiency, because the initial concentration provided a strong driving force viz. the concentration gradient to overcome the mass transfer resistance between the aqueous phase and the solid [1,26]. In the present study, the same trend was found. In Figure 7, the effect of the initial EBT concentration on the adsorption process with F-SAC is shown. It can be observed that during the early phase, the adsorption rates were the most rapid for each of the initial EBT concentrations. As the binding sites on the F-SAC surface were gradually occupied, the EBT removal rate decreased due to the limited adsorption sites.

Moreover, the lower the initial EBT concentration, the shorter the time that was needed to reach adsorption equilibrium. When the initial EBT concentration was 100 mg·L^−1^ or 300.0 mg·L^−1^, the time it took to reach adsorption equilibrium was around 720 min. When the initial EBT concentration was increased to 600 mg·L^−1^, the adsorption equilibration time became 2400 min. As the initial EBT concentration was increased to 1000 mg·L^−1^, the adsorption capacity reached saturation, and the final adsorption capacity was 240.97 mg·g^−1^.

Table 3 lists the binding capacity and binding affinity of the Langmuir and Freundlich models for both F-SAC and P-SAC. In general, both models could be used to describe the EBT adsorption process. The Langmuir model assumes a fixed number of accessible sites on the adsorbent surface, and the number of molecules adsorbed and desorbed on a unit surface in a unit time is equal [17]. The Freundlich model is an empirical equation that assumes a heterogeneous adsorbent surface with adsorption sites at varying energy levels [2]. The EBT adsorption behavior was affected by the interactions between the solution environment and the imperfect F-SAC crystal surface, thus it should belong mostly to the multilayer type of adsorption. As can be seen from Table 3, the adsorption capacity of P-SAC was only about half that of the F-SAC, and the adsorption process of P-SAC fit the Langmuir model more closely.

#### 3.2.3. Adsorption Kinetic Studies

Through the study on adsorption kinetics, the rates of adsorption were compared and the rate-limiting step was determined. In addition, it was helpful in deducing the adsorption mechanism. Thus information on the adsorption kinetics was essential for selecting proper operating conditions [2,24]. 

**Figure 8:**
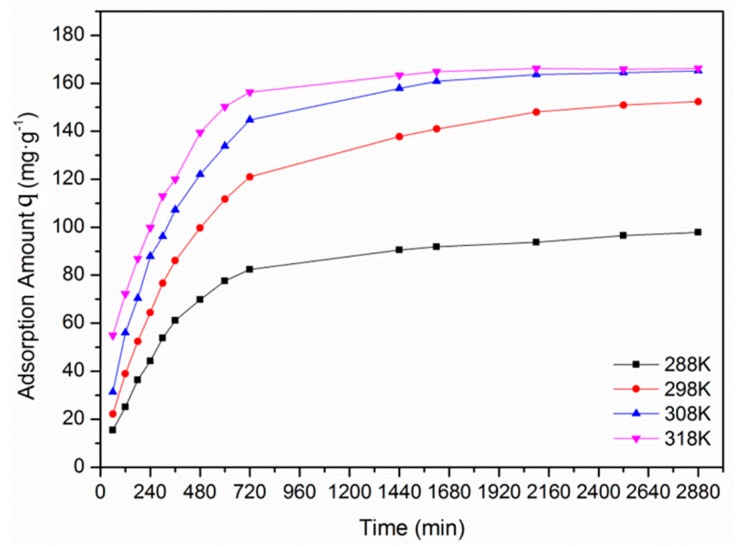
The effect of temperature on the adsorption of EBT by F-SAC.

Figure 8 shows the plot of EBT adsorption by F-SAC versus time at different temperatures, and these data were fitted by Lagrangian first-order model and pseudo-second-order reaction model, the calculation results were summarized in Table 4. It shows that the correlation coefficients for both the Lagrangian first-order model and the pseudo-second-order model are high. However, the correlation coefficients for the pseudo-second-order model are slightly higher than those of the Lagrangian first-order model under all four investigated temperatures, and the theoretical q_e_ values of the pseudo-second-order model were closer to the experimental values, which means the pseudo-second-order model is more applicable for analyzing the EBT adsorption process.

From the fact that EBT adsorption followed pseudo-second-order kinetics better, it was reasonable to conclude that boundary layer resistance was not the rate-limiting step, thus the adsorption rate might be controlled mainly by chemical processes that involve valency forces through sharing or exchanging of electrons between EBT and SAC [24,27,28], although physical adsorption co-occurred.

From Table 4 it is not hard to see that from 288 to 318 K, the experimental q_e_ increased with temperature and achieved its maximum at the temperature of 318 K, due to the decreased solution viscosity and increased EBT mobility. Further increasing the temperature did not bring rapid q_e_ improvement, which could be attributed to decreased surface activity, that is to say the active binding sites were damaged and the binding forces between the EBT molecules and the SAC were weakened, thus the rate of increase of q_e_ became slow [29,30].

#### 3.2.4. Thermodynamic Analysis

Since the EBT elimination by SAC was related to temperature, a thermodynamic analysis was conducted to understand the influence of temperature. The thermodynamic parameters of standard entropy change ΔS (J·mol^−1^·K^−1^), standard enthalpy change ΔH (kJ·mol^−1^), and standard free energy change ΔG (kJ·mol^−1^) could be obtained from sorption isotherms using Equations (7) and (8), where D = q_e_/c_e_, T is absolute temperature (K), and R (8.314 J·mol^−1^·K^−1^) is the ideal gas constant [31]:

(7) $$\mathrm{lnD}=\frac{\Delta S}{R}-\frac{\Delta H}{\mathrm{RT}}$$

(8) ΔG=ΔH−TΔS

The calculated thermodynamic parameters were recorded in Table 5. The positive ΔH value indicates the endothermic nature of the adsorption process. The positive ΔS value demonstrates the process was entropy-driven, suggesting improved randomness at the solid-liquid interface during the EBT adsorption process, thus the EBT-SAC system shows van der Waals interactions [31,32]. The negative ΔG values imply that the EBT adsorption on SAC was thermodynamically favorable. The value of ΔG became more negative as the temperature increased, indicating increasingly favorable adsorption in higher-temperature conditions [31]. From this analysis it can be concluded that the adsorption process for EBT entrapment onto SAC is naturally endothermic and spontaneous.

## 4. Conclusions

In this study, sewage sludge was activated by Fenton’s reagent and calcinated to make SAC. The experimental results showed that the produced SAC had a porous structure and high specific surface area due to uniform and abundant tiny pores. Graphite-like structures of carbon were formed in the produced SAC, and abundant functional groups were formed on its surface

The prepared SAC samples were used as adsorbents for EBT dye removal from water and exhibited excellent adsorption performance. Through adsorption isotherm, kinetic, and thermodynamic studies, it was found that the adsorption capacity of SAC was greatly improved by Fenton reagent activation. Increasing the initial concentration of EBT could increase its removal efficiency by SAC. The adsorption capacity also increased with temperature, with the highest adsorption capacity reaching 178.2 mg·g^−1^ in 48 h at the temperature of 318 K and pH 6. The adsorption of EBT onto Fenton-activated SAC was naturally endothermic and spontaneous, belonged mostly to the multilayer type of adsorption, and involves both physical and chemical processes. 

## Figures and Tables

**Table 1:** Properties of the sewage sludge.

Item	Analysis Results
Sewage sludge	
pH	6.8–8.1
Water content (%)	98.6–99.4%
Total solid (g·L^−1^)	17.63
Volatile solid (g·L^−1^)	14.01
Element composition of dried sewage sludge	
C (%)	36.01
H (%)	5.09
N (%)	6.22
S (%)	1.13

**Table 2:** Properties of the SACs.

Properties	F-SAC	P-SAC
Brunauer-Emmett-Teller (BET) surface area (m^2^·g^−1^)	172.8	92.6
Pore volume (cm^3^·g^−1^)	0.196	0.099
S_micro_ (m^2^·g^−1^)	10.0	35.13
V_micro_ (cm^3^·g^−1^)	4.33 × 10^−3^	1.11 × 10^−2^
Average pore diameter (nm)	4.54	4.28

**Table 3:** Langmuir and Freundlich correlations for the adsorption process.

Models		F-SAC	P-SAC
Freundlich	equation	y = 0.2234x + 1.8327	y = 0.0379x + 1.8497
	K_F_	68.03	70.75
	1/n	0.2234	0.0379
	R^2^	0.9483	0.7717
Langmuir	equation	y = 4.579 × 10^−3^x + 0.0367	y = 0.0101x + 0.2233
	K_L_	0.1247	0.04523
	q_m_	218.4	99.01
	R^2^	0.9196	0.9883

**Table 4:** Kinetic constants for EBT adsorption by F-SAC.

**Lagrangian First-Order Model**					
T (K)	equation	k_1_ (min^−1^)	q_e_ (mg·g^−1^)	R^2^	Experimental q_e_ (mg·g^−1^)
288	y = −1.620 × 10^−3^x + 4.290	1.620 × 10^−3^	72.95	0.9838	96.9
298	y = −1.645 × 10^−3^x + 4.727	1.645 × 10^−3^	112.9	0.9827	153.4
308	y = −2.115 × 10^−3^x + 4.880	2.115 × 10^−3^	131.6	0.9932	165.1
318	y = −2.866 × 10^−3^x + 4.797	2.370 × 10^−3^	121.2	0.9329	178.2
**Pseudo-Second-Order Model**					
T (K)	equation	k_2_ (g·mg^−1^·min^−1^)	q_e_ (mg·g^−1^)	R^2^	Experimental q_e_ (mg·g^−1^)
288	y = 9.151 × 10^−3^x + 3.063	1.734 × 10^−5^	109.3	0.9979	96.90
298	y = 5.827 × 10^−3^x + 1.825	1.861 × 10^−5^	171.6	0.9976	153.4
308	y = 5.494 × 10^−3^x + 1.370	2.203 × 10^−5^	182.0	0.9988	165.1
318	y = 5.653 × 10^−3^x + 0.8385	3.811 × 10^−5^	176.9	0.9988	178.2

**Table 5:** Thermodynamic parameters for EBT adsorption by F-SAC.

ΔH (kJ·mol^−1^)	ΔS (J·mol^−1^·K^−1^)	ΔG (kJ·mol^−1^)			
		288 K	298 K	308 K	318 K
143.4	492.4	−1.586	−3.338	−8.262	−13.186
